# Obstructive sleep apnea is position dependent in young infants

**DOI:** 10.1038/s41390-022-02202-9

**Published:** 2022-08-16

**Authors:** Hanna-Leena Kukkola, Turkka Kirjavainen

**Affiliations:** 1 Department of Pediatrics, New Children’s Hospital, Helsinki, Finland; 2 Pediatric Research Center, New Children’s Hospital, Helsinki University Hospital, Helsinki, Finland; 3 Children’s Hospital Department of Clinical Neurophysiology and Neurological Sciences, HUS Medical Imaging Center, Helsinki University Central Hospital, Helsinki, Finland

## Abstract

**Background:**

Obstructive sleep apnea in infants with Pierre Robin sequence is sleep-position dependent. The influence of sleep position on obstructive events is not established in other infants.

**Methods:**

We re-evaluated ten-year pediatric sleep center data in infants aged less than six months, with polysomnography performed in different sleep positions. We excluded infants with syndromes, genetic defects, or structural anomalies.

**Results:**

Comparison of breathing between supine and side sleeping positions was performed for 72 infants at the median corrected age of 4 weeks (interquartile range (IQR) 2-8 weeks). Of the infants, 74% were male, 35% were born prematurely, and 35% underwent study because of a life-threatening event or for being a SIDS sibling. Upper airway obstruction was more frequent (obstructive apnea-hypopnea index (OAHI), *p* < 0.001), 95th-percentile end-tidal carbon dioxide levels were higher (*p* = 0.004), and the work of breathing was heavier (*p* = 0.002) in the supine than in the side position. Median OAHI in the supine position was 8 h^−1^ (IQR 4–20 h^−1^), and in the side position was 4 h^−1^ (IQR 0-10 h^−1^).

**Conclusions:**

Obstructive upper airway events in young infants are more frequent when supine than when sleeping on the side.

**Impact:**

- The effect of sleep position on obstructive sleep apnea is not well established in infants other than in those with Pierre Robin sequence.
- A tendency for upper airway obstruction is position dependent in most infants aged less than 6 months. Upper airway obstruction is more common, end-tidal carbon dioxide 95th-percentile values higher, and breathing more laborious in the supine than in the side-sleeping position.
- Upper airway obstruction and obstructive events have high REM sleep predominance.
- As part of obstructive sleep apnea treatment in young infants, side-sleeping positioning may prove useful.

## Introduction

Infants commonly experience single obstructive events during sleep.^1–3^ However, severe adult-type obstructive sleep apnea (OSA) is rare and often related to syndromes such as Pierre Robin sequence (PRS).^1,3,4^ Obstructive events in infants show REM-sleep dominancy, and they do not commonly interrupt sleep to the same degree as in adults.^1^ Gastroesophageal reflux appears to lead to increased numbers of obstructive events, although the reflux and apnea episodes do not generally appear simultaneously.^5^ The significance of OSA in infants is not yet established. Obstructive events have been associated with increased risk for sudden infant death syndrome (SIDS).^6,7^ Currently, no consensus exists: neither as to criteria for degree of upper airway obstruction should be considered OSA nor when OSA should be treated.^1^

OSA is sleep position dependent in adults, older children and in infants with PRS.^4,8–10^ Only one polysomnography (PSG) -based study concerns the effect of sleep position on upper airway obstruction in infants without any syndrome. Orr and associates^11^ showed in 65 infants that upper airway obstruction was neither position- nor sleep-stage dependent. However, in their study the detection of airflow detection was not based on detection of reliable flow signal, but on carbon-dioxide signal.

After recognition of position dependency of OSA in infants with PRS^4^ and preliminary observations in other infants, we included position control in our infant PSG protocol and, whenever upper airway obstruction was suspected or observed, we performed PSG in supine and side-sleep positions. The aim of this study was to determine the effects of sleep position on upper airway obstruction in infants less than six months of age.

## Methods

### Study design and patients

**Fig. 1:**
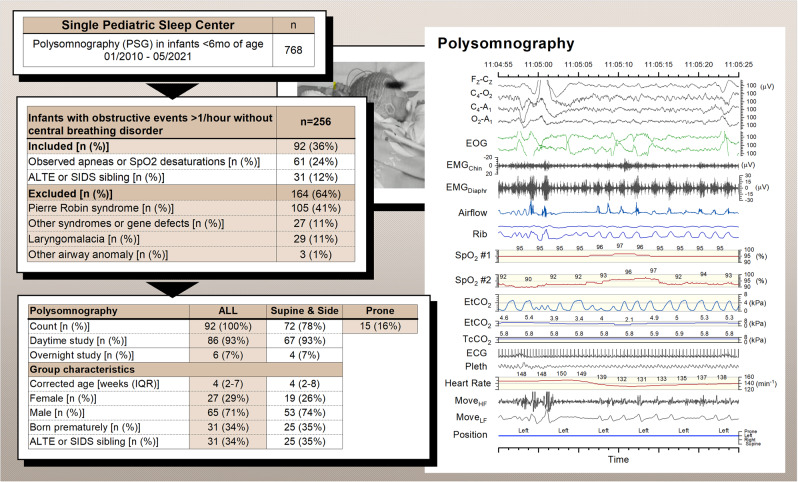
Study flow chart, infant characteristics, and polysomnography setup. ALTE apparent life-threatening event, ECG electrocardiogram, EEG electroencephalogram, EMG electromyogram, EOG electro-oculogram, EtCO_2_ end-tidal carbon dioxide, IQR interquartile range, Move_HF_ movement sensor high-frequency band representing general movements, Move_LF_ movement sensor low-frequency signal representing respiratory movements, Pleth oximeter plethysmography, Rib respiratory induction plethysmography, SIDS sudden infant death syndrome, SpO_2_ pulse oximeter oxyhemoglobin saturation, TcCO_2_ transcutaneous carbon dioxide.

We evaluated our pediatric sleep unit data from 2011 to 2021, including infants less than six months old showing obstructive events but without predominance of central breathing disorders and periodic breathing in PSG (Fig. 1). We excluded infants with recognized facial and upper airway abnormalities, syndrome, or genetic abnormalities. The Helsinki University ethics committee (HUS/3186/2019) and New Children’s Hospital Institutional Review Board (Project #4980) approved the study protocol.

We performed sleep studies in supine and side positions if obstructive events were suspected or observed during online PSG analysis. Infants were also placed in a prone position on a few occasions. At least one cycle of rapid-eye-movement sleep (REM) and of non-REM sleep was recorded in each position. The sleep studies took place during daytime in infants less than three months old, and during night-time in older infants.

### Polysomnography

The PSG setup and analysis followed the recommendations of the American Academy of Sleep Medicine (AASM).^12^ We used Embla N700 (Natus Medical Inc., WI) prior to 2018, and the SOMNO^TM^ HD (SOMNOmedics GmbH, Germany) PSG systems thereafter. The PSG protocol (Fig. 1) comprised monitoring of four electroencephalogram channels (Cz-Fz, Cz-O2, C4-M1, O2-M1), two electro-oculography channels, chin- and diaphragm electromyography (EMG), nasal airflow by pressure transducer, two respiratory inductive plethysmography belts, electrocardiography, two pulse oximeters (SpO_2_) with a two- to-four second averaging interval (Embla or SOMNO^TM^ HD, and Masimo Radical Pulse CO-Oximeter, Masimo Co, CA), end-tidal carbon dioxide (EtCO_2_) (CAP10 Capnograph, Medlab medizinische Diagnosegeräte GmbH, Germany), transcutaneous carbon dioxide (TcCO_2_) (SenTec Inc., MO), a movement sensor mattress (Emfit Ltd, Finland), a position sensor, and a synchronized video recording. Thermistor was added to the setup if oral breathing was suspected or noted.

All PSG recordings we reanalyzed (T.K.) blinded to position, and we visually scored the sleep stage and respiratory events. Central, obstructive, and mixed apneas, and obstructive hypopneas were recognizable based on airflow, diaphragm EMG activity, and respiratory movement. Work of breathing (WOB) we estimated from the diaphragm EMG activity and graded from 0 to 2, where 0 represented normal, 1 slightly increased inspiratory EMG activity, and 2 laborious breathing with clearly increased activity (Supplementary Fig. S1). We performed WOB scoring twice in a blinded manner with an over six-month time interval between scoring sessions to calculate inter-scorer (T.K.) repeatability (Supplementary Table S1). Oxygen desaturations over 3% (ODI_≥3_) were analyzed both separately and in relation to the preceding apnea or hypopnea (ODI_≥3_ central apnea / obstructive apnea/obstructive hypopnea/mixed apnea). We used self-developed (T.K.) special purpose software to complete detailed SpO_2_, TcCO_2_ analyses, and breath-by-breath analyses of EtCO_2_ and of breathing frequency. Software allowed us to compare combined-event data such as over 3% desaturation events (OD_≥3_) in relation to obstructive, mixed, and central apneas and obstructive hypopneas.

### Statistical methods and analysis

The statistical analysis required IBM® SPSS® Statistics software version 25 and OriginPro 2022. Because PSG parameters were not normally distributed, we selected non-parametric tests for the analysis. The Wilcoxon signed-rank test served for the pairwise comparison between sleep positions and sleep stages. We used Mann-Whitney Test to compare the changes in breathing parameter results between supine and side sleeping positions in the two groups of infants with different order of sleeping position. Kappa statistics served to calculate repeatability of WOB scoring. The level of significance we set at *p* < 0.05.

## Results

During the ten-year study period, a total of 786 PSG studies took place with infants less than six months of age. After the application of our exclusion criteria, a total of 92 infants presented with obstructive events, and 72 were eligible for comparison between supine and side-sleeping positions. Because these positions were preferable sleeping positions, were therefore placed only 15 infants in a prone position (See Supplementary Fig. S2). The median corrected age of 72 infants was 4 weeks (IQR 2-8 weeks), with 53 (74%) male, 25 (35%) were born prematurely, and another 25 (35%) were studied because of an earlier apparently life-threatening event (ALTE) or because each was the sibling of an infant who had died from SIDS (Fig. 1). Findings for these two subgroups: the premature infants and the ALTE infants or SIDS-siblings, were similar to those for the rest of infants.

Median PSG recording time was 4.0 h (IQR 3.2–4.7 h) and total sleep time 2.4 h (IQR 2.0–2.9 h). Out of the 72 infants who slept both in supine and in side positions, 55 (76%) were placed first in the supine position. The groups with different sleep position order had similar changes in number of apneas, hypopneas and oxygen desaturations events (*p* = 0.16 – 0.68).

**Fig. 2:**
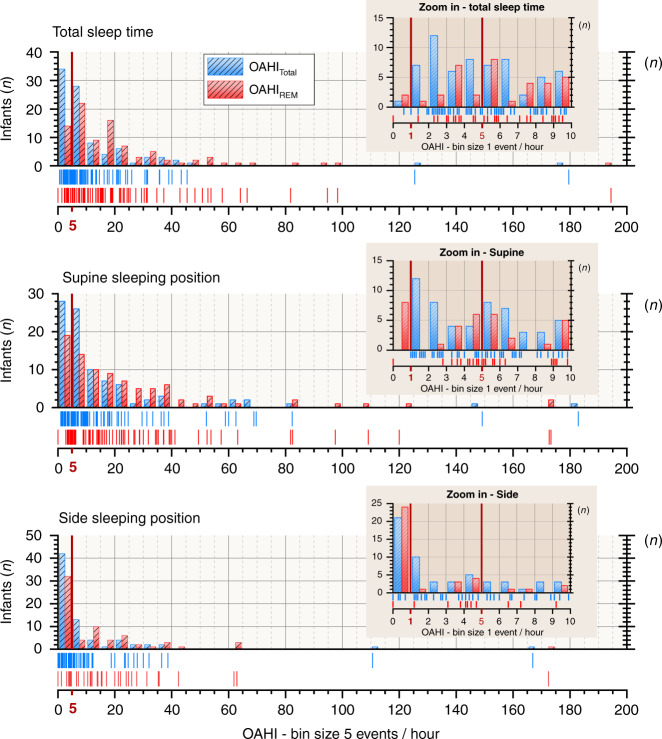
Obstructive events during sleep. Number of infants according to obstructive apnea-hypopnea index (OAHI). Red vertical lines indicate OAHI levels of 1 (zoomed figures) and 5 per hour during sleep. OAHI shifts from higher values towards lower, normal values from supine- to side-sleeping position. In supine, all infants (72) had OAHI above 1 per hour and 48 (67%) had above 5 per hour of sleep. In the side-sleeping position, 51 (71%) infants had OAHI above 1 per hour and 30 (42%) had above 5 per hour during sleep.

Most of these infants experienced with few obstructive events during our sleep study, but all experienced obstructive events at the rate of at least one per hour of sleep when supine (Fig. 2). Of the infants, 58 (63%) had an obstructive apnea-hypopnea index (OAHI) over 5 per hour.

**Fig. 3:**
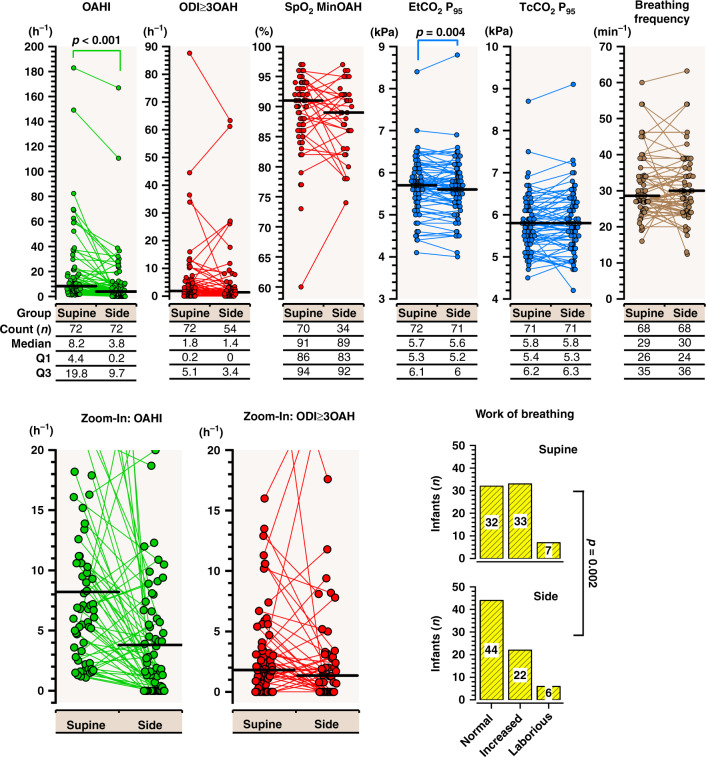
Obstructive breathing events and work of breathing. Side sleeping position reduced the number of obstructive events, and of end-tidal carbon dioxide 95th percentile levels, and in 13 (18%) infants, reduced work of breathing when compared to values in a supine-sleeping position. EtCO_2_P_95_ 95th-percentile level of end-tidal carbon dioxide, OAHI obstructive apnea-hypopnea index, ODI_≥3_OAHI oxyhemoglobin desaturations index of ≥3% from baseline following obstructive and mixed apneas, or obstructive hypopneas, SpO_2_ MinOAHI minimum pulse oximeter hemoglobin saturation value following obstructive and mixed apneas, or obstructive hypopneas, TcCO_2_P_95_ 95th-percentile level of transcutaneous carbon dioxide.

**Fig. 4:**
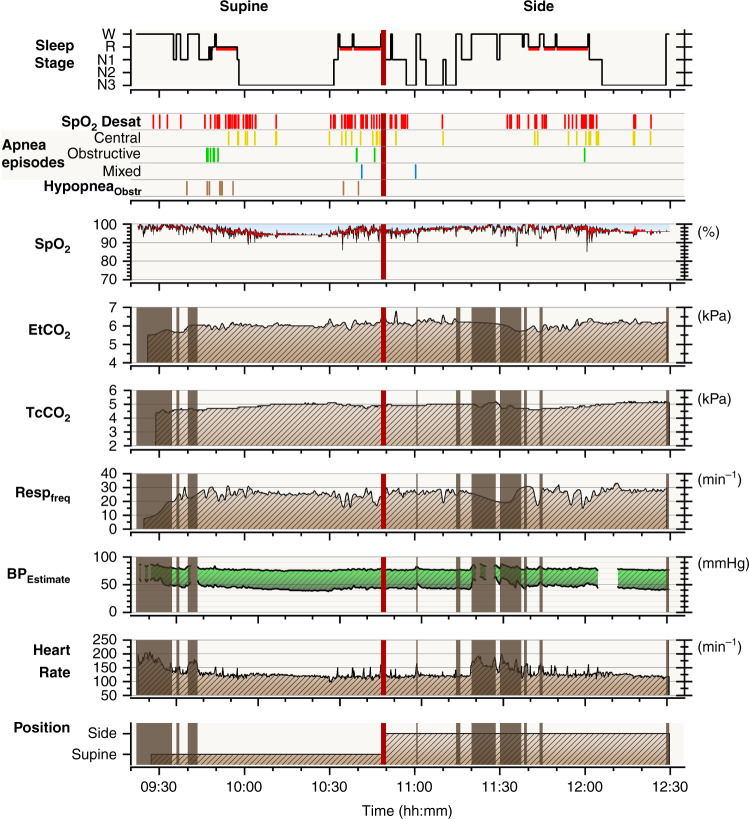
Polysomnography findings. Recording summary of a two-month-old infant with position dependent obstructive sleep apnea. The infant first slept supine followed by recording in a side position. Obstructive and mixed apneas occurred almost entirely in the supine-sleeping position during REM sleep. In the second horizontal panel, one vertical bar presents one event. Brown vertical shadow bars cover periods of wakefulness with unreliable signal recording. BP blood pressure. Values calculated from pulse transit time. EtCO_2_ end-tidal carbon dioxide, Resp_freq_ breathing frequency, SpO_2_ Desat pulse oximeter oxyhemoglobin desaturation ≥3%, TcCO_2_ transcutaneous carbon dioxide.

The appearance of upper airway obstruction was both sleep-position and sleep-stage dependent (Table 1, Figs. 2–4). OAHI was higher in the supine than on the side position (*p* < 0.001) and in REM than in non-REM sleep (*p* < 0.0001). Estimated WOB was more laborious in 13 (18%) infants (*p* = 0.01), but EtCO_2_ 95th-percentile values were systematically higher (*p* = 0.004) on the supine than on the side position. Only one infant (1%) experienced with heavier WOB in the side than in the supine position. Most of the oxygen desaturations observed in these infants were related to central apneas, and sleep position did not affect their appearance (Table 1).

**Table 1:** Sleep and breathing events.

	Supine			*p* value
Parameters	All infant	Included in comparison	Side	Supine vs side
Number of infants	92	72	72	
Sleep characteristics				
Recording Time (min)	106 (73–162)	92 (68–129)	64 (41–101)	0.001
Total sleep time (min)	70 (52–114)	63 (50–87)	51 (33–77)	0.02
Sleep efficiency (%)	71 (59–81)	71 (57–86)	81 (66–90)	0.005
Time in non-REM Sleep (min)	46 (34–77)	42 (34–62)	32 (24–50)	0.01
Tim in REM Sleep (min)	22 (14–39)	19 (13–31)	15 (9–30)	0.16
Breathing characteristics				
OAHI (hour^−1^)	8 (4–18)	8 (4–20)	4 (0–10)	<0.001
OAHI_REM_ (hour^−1^)	16 (6–35)	19 (6–36)	8 (0–21)	<0.001
OAHI_non-REM_ (hour^−1^)	3 (1–10)	3 (1–10)	1 (0–4)	<0.001
OAI (hour^−1^)	3 (2–8)	4 (2–10)	0 (0–4)	<0.001
CAI (hour^−1^)	6 (3–11)	6 (2–11)	9 (1–15)	<0.05
MAI (hour^−1^)	2 (1–4)	2 (1–4)	1 (0–5)	<0.05
ODI_≥3_ (hour^−1^)	6 (3–20)	7 (3–21)	13 (3–24)	0.37
ODI_≥3OAH_ (hour^−1^)	2 (0–4)	2 (0–5)	1 (0–4)	0.08
SpO_2 MinOAH_ (%)	91 (87–94)	91 (87–94)	90 (83–92)	0.48
SpO_2 Median_ (%)	98 (97–99)	98 (97–99)	98 (96–99)	0.002
EtCO_2_ P_95_ (kPa)	5.7 (5.4–6.1)	5.7 (5.2–6.1)	5.6 (5.2–6.0)	0.004
TcCO_2_ P_95_ (kPa)	5.8 (5.4–6.2)	5.8 (5.4–6.2)	5.9 (5.3–6.3)	0.17
Work of breathing (0 to 2)	0.7 (0–1)	0.5 (0–1)	0.5 (0–1)	0.01
Breathing Frequency (min^−1^)	28 (25–34)	29 (25–36)	30 (24–36)	0.98

Results are presented as median (interquartile range, (IQR)), except for work of breathing presented as mean (IQR) at a scale of 0 (normal), 1 (increased), and 2 (laborious).

*CAI* central apnea index; *EtCO*_*2*_*P*_*95*_ 95^th^-percentile level of end-tidal carbon dioxide, *MAI* mixed apnea index, *Non-REM* non-rapid-eye-movement, *OAHI* obstructive apnea-hypopnea index, *OAI* obstructive apnea index, *ODI*_*≥3 OAH*_ pulse oximeter desaturation index of ≥3% following obstructive and mixed apneas, or obstructive hypopneas, *REM* rapid-eye-movement, *SpO*_*2 MinOAH*_ pulse oximeter minimum oxyhemoglobin saturation following obstructive and mixed apneas, or obstructive hypopneas, *SpO*_*2 Median*_ pulse oximeter oxyhemoglobin saturation median value, *TcCO*_*2*_*P*_*95*_ 95^th^-percentile level of transcutaneous carbon dioxide.

## Discussion

We have shown in 72 infants aged less than six months that the appearance of upper airway obstruction is consistently both sleep-position and sleep-stage dependent. Obstructive events were more common, end-tidal carbon dioxide 95th-percentile levels higher, and breathing was more laborious in the supine than in the side position. As well established, obstructive events here were also much more common in REM than in non-REM sleep.

### OSA and sleep position

In children and adults, despite the fact that upper airway narrowing and increased tendency for upper airway collapse have an underlying structural cause, OSA is often sleep-position dependent. OSA is most often more severe in the supine than in the side- or prone-sleeping positions.^8,9^ The same position dependency it true for young infants with PRS and small chin,^4,10^ but also, as shown in our study, is true for most infants with OSA even without any clear predisposing structural deficit. The anatomical sites of upper airway obstruction in young infants are similar to sites in adults with OSA.^13^

OSA during the first three months seems to have its own characteristics, and as in our study cohort, it may appear without any noticeable anatomical abnormality.^13^ OSA in young infants is evident especially in young infants with gastroesophageal reflux,^5,14,15^ in premature infants,^16–19^ and in infants with earlier ALTE.^7,20^ Regardless of the underlying condition, infant OSA seems to be resolved by the age of four to eight months.^4,21–23^

In a study by Pereira et al.^24^, 50 infants older than ours, aged 8 to 12 months with mild OSA, showed no significant effect of sleep position on OSA. In children with normal facial structure who were older than six months, OSA has been most closely related to adenoidal or tonsillar hypertrophy.^17,25^ This difference in OSA etiology is also likely to explain the differing findings between younger and older infants. In our study group, clinical and PSG follow-up showed the resolution of obstructive sleep apnea in most of our infant before they reached 4–8 months old.

Most of our infants had mild OSA if compared, for example, to the OSA observed in many PRS infants.^4^ All of ours had a normal baseline SpO_2_ level, and the great majority also had normal EtCO_2_ and TcCO_2_ levels, together with a low number of oxygen desaturations in related to obstructive and mixed apneas and obstructive hypopneas. Approximately half of our infants displayed normal work of breathing while in the supine-, and two-thirds while in the side-sleeping position. The reduction in 95th-percentile levels of EtCO_2_ from supine- to side position may reflect a resolution of partial upper airway obstruction, but no cause for this reduction could be unequivocally determined.

The effect of sleep positioning observed here may not be considered sufficient to treat OSA in some infants. However, side-position for sleep may still prove useful as accessory to other forms of treatment such as high-flow nasal cannula (HFNC) or continuous positive airway pressure (CPAP).

### Role of sleep positioning in infants

Currently, the supine sleep position is recommended for all infants as part of SIDS-reduction campaigns.^26^ No exceptions attract attention, despite infants such as those with PRS seeming to benefit from the side-sleep positioning.^4^ One estimated odds ratio (OR) for SIDS between the supine and prone sleeping position is 4.46, and is 1.36 between supine and side sleeping positions.^27^ How real is the risk of SIDS being related to side-sleep positioning if side or prone positioning were applied as a part of OSA treatment? Prior to SIDS sleeping campaigns in the 1970’s and 1980’s, SIDS rates in Finland ranged from 0.5 to 0.6 per 1000 live births. In order to establish the safety of any type of OSA treatment, the study group should exceed 7 600 infants when comparing treatment safety to sleep position treatment with prone sleep positioning, and over 76,000 for comparing the treatment to side-sleep positioning. This simple power calculation demonstrates the low risk level related to side sleep positioning. It may not be valid to apply the standard sleep position recommendation to a specific infant population from whom side-sleep positioning may be otherwise beneficial.^4^

### Study limitations

Here, most of our PSG studies were done as daytime recording for clinical purposes. The recordings were approximately half of the length as obtainable by standard overnight PSG. The recordings were continued in each sleeping position as long as needed for appropriate clinical decision-making, each recording at least one full sleep cycle including non-REM and REM periods in each position. Daytime recordings have their pros and cons, but generally the literature supports their clinical use in infants age less than three months.^3,28,29^ In this age group, infants generally sleep enough during daytime for reliable PSG analysis.^30,31^ In Finland, daytime recordings are more readily available than whole-night recordings. Daytime recordings allowed a pediatric pulmonologist’s online surveillance of PSG if indicated. Thus, for example our study group, in the majority of cases, PSG studies were continued until proper clinical decision-making was possible in respect to sleep position. Despite the time limitations, we do not conclude that whole-night PSG recording would have significantly affected the study conclusions.

We used diaphragm EMG for a rough estimate of breathing effort. EMG was recorded by surface electrodes, and measurement amplitude was compared to baseline levels during quiet breathing, and to baseline background activity. Such a measurement is difficult to calibrate properly and is affected by signal filtration and, impedance, and by sleep stage (Supplementary Fig. S1).^32^ We did not have automatic breath-by-breath analysis of diaphragm EMG similar to what we had for EtCO_2_ and breathing frequency analysis. A proper estimation of breathing effort would have required esophageal pressure measurement,^13,33^ and diaphragm and esophageal pressure measurement are expected to show only modest correlation.^34^ Despite these limitations, we find diaphragm EMG useful, because no readily available alternatives exist.^4^ Our WOB scoring showed good repeatability (Supplementary Table S1), and we believe the scoring reflects the real WOB changes between sleep positions with reasonable accuracy.

## Conclusions

In most infants without any noticeable structural facial or airway anomaly or syndrome, the upper airway obstruction was both sleep-position and sleep-stage dependent, with the side-sleep position reducing the frequency of obstructive events when compared with the supine position.

## Supplementary information

Supplementary Materials

## Data Availability

The datasets generated during and/or analyzed during the current study are available from the corresponding author on reasonable request.
