# Supplementary material for: Obstructive sleep apnea is position dependent in young infants
Source: Pediatr Res. 2022 Aug 16;93(5):1361–7. doi: 10.1038/s41390-022-02202-9 (PMC10132964; doi:10.1038/s41390-022-02202-9)
Supplement: Supplementary file 1 — Supplementary Materials [file 41390_2022_2202_MOESM1_ESM.pdf]

1. **Title:** Obstructive sleep apnea is position dependent in young infants

2. **Author listing:** Hanna-Leena Kukkola[1,2], Turkka Kirjavainen[1,2,3]

3. **Name of departments and institutions:** [1]Department of Pediatrics, New Children's Hospital, Helsinki, Finland, [2]Pediatric Research Center, New Children's Hospital, Helsinki University Hospital, Helsinki, Finland; [3]Children's Hospital Department of Clinical Neurophysiology and Neurological Sciences, HUS Medical Imaging Center, Helsinki University Central Hospital, Helsinki, Finland

Supplementary data:

Supplementary Fig. S1: Scoring of work of breathing.

Supplementary Table S1: Intra-scorer repeatability of work of breathing. Kappa statistics.

Supplementray Fig. S2**:** Obstructive breathing events and work of breathing.

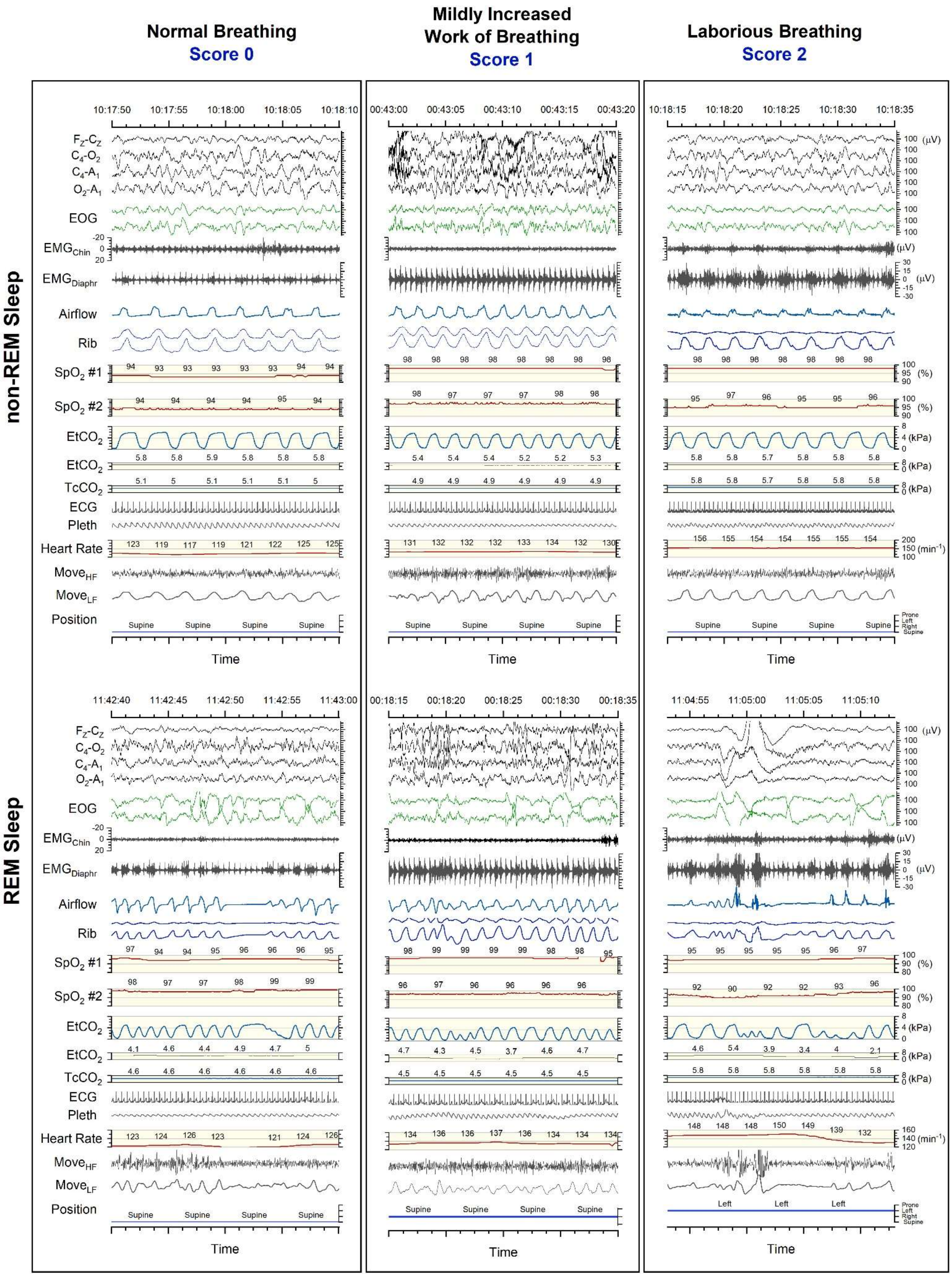

Supplementary Fig. S1 Scoring of work of breathing (WOB). (continued on the next page…)

(…continued from the previous page)

WOB scoring was performed from diaphragm EMG signal. Figure presents example recordings of WOB score in both non-REM and REM sleep in each scoring category 0-2: (0) normal breathing, (1) mildly increased inspiratory diaphragm EMG activity and work of breathing, and (2) laborious breathing with clearly increased diaphragm EMG activity.

ECG electrocardiogram, *EEG* electroencephalogram, *EMG* electromyogram, *EOG* electro-oculogram, $EtCO_2$ end-tidal carbon dioxide, $Move_{HF}$ movement sensor high-frequency band representing general movements, $Move_{LF}$ movement sensor low-frequency signal representing respiratory movements, *Pleth* oximeter plethysmography, *REM* rapid-eye-movement, *Rib* respiratory induction plethysmography, SpO_2 pulse oximeter oxyhemoglobin saturation, $TcCO_2$ transcutaneous carbon dioxide.

Supplementary Table S1. Intra-scorer repeatability of work of breathing (WOB) score presented both as a figure and a cross-table. WOB score was based on diaphragm EMG signal (Supplementary Fig. S1) and WOB was classified as (0) normal, (1) mildly increased inspiratory EMG activity and work of breathing, and (2) laborious breathing with clearly increased diaphragm EMG activity. We scored WOB twice in 72 infants separately in supine and side sleeping positions (2 x 72 = 144 comparisons) on a blinded manner at six-month time interval between the two scoring sessions. Double scoring showed reasonable repeatability with Kappa-value of 0.63 (confidence interval 0.52-0.75, $p < 0.001$). There was no overlapping between the WOB categories of (0) normal and (2) laborious breathing.

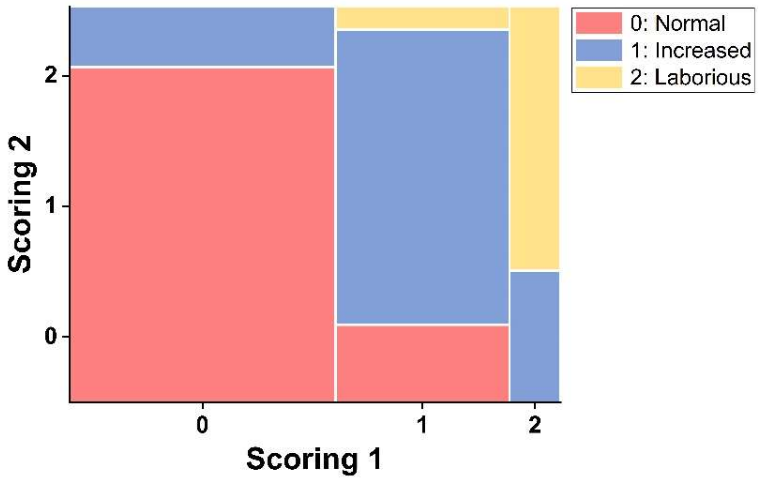

		Scoring 2: WOB category (count)			
		0	1	2	Total count
Scoring 1: WOB category (count)	0	66	12	0	78
	1	10	38	3	51
	2	0	5	10	15
Total count		76	55	13	144

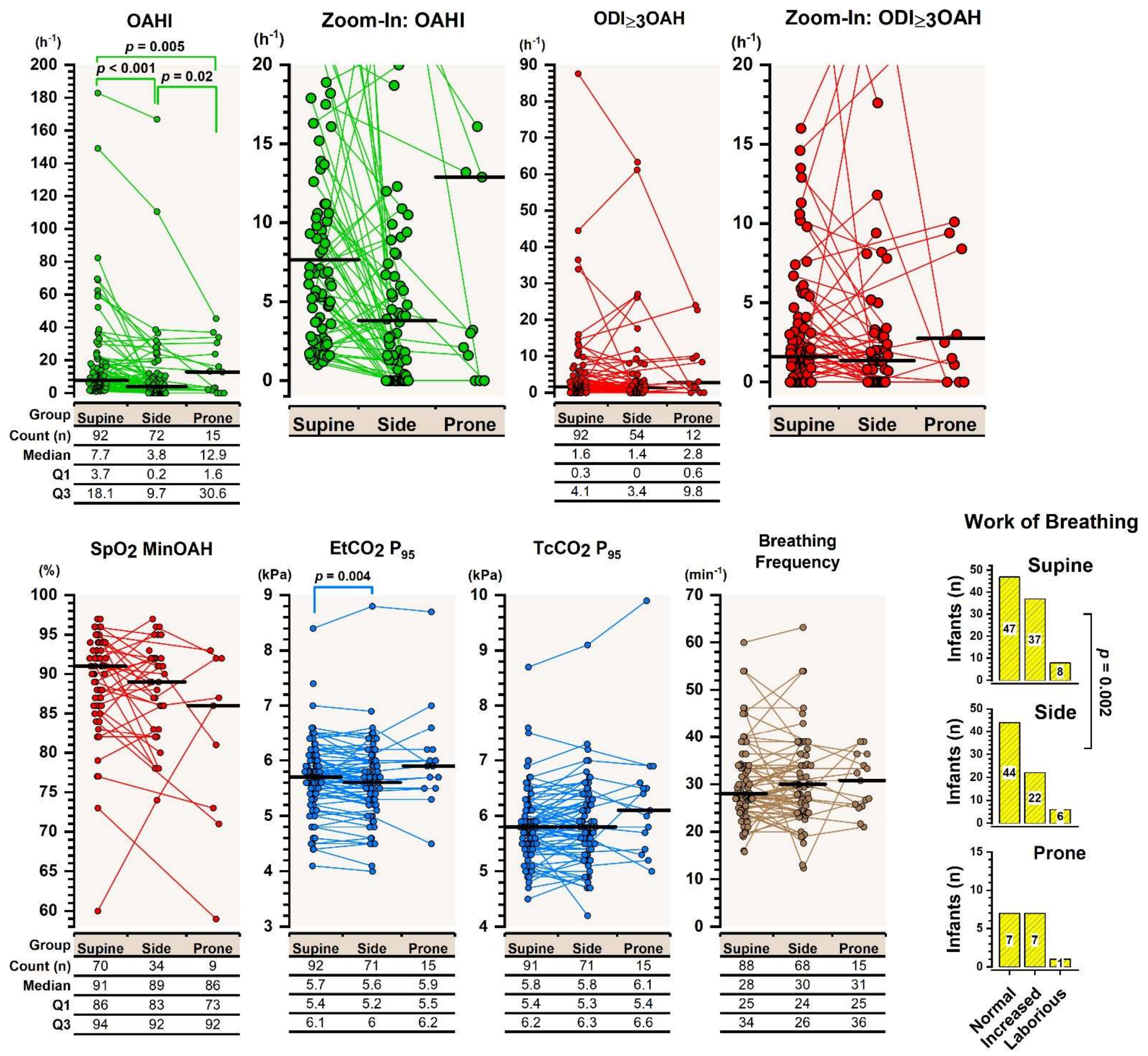

OAHI

Group	Supine	Side	Prone
Count (n)	92	72	15
Median	7.7	3.8	12.9
Q1	3.7	0.2	1.6
Q3	18.1	9.7	30.6

ODI≥3OAH

Group	Supine	Side	Prone
Count (n)	92	54	12
Median	1.6	1.4	2.8
Q1	0.3	0	0.6
Q3	4.1	3.4	9.8

SpO2 MinOAH

Group	Supine	Side	Prone
Count (n)	70	34	9
Median	91	89	86
Q1	86	83	73
Q3	94	92	92

EtCO2 P95

Group	Supine	Side	Prone
Count (n)	92	71	15
Median	5.7	5.6	5.9
Q1	5.4	5.2	5.5
Q3	6.1	6	6.2

TcCO2 P95

Group	Supine	Side	Prone
Count (n)	91	71	15
Median	5.8	5.8	6.1
Q1	5.4	5.3	5.4
Q3	6.2	6.3	6.6

Breathing Frequency

Group	Supine	Side	Prone
Count (n)	88	68	15
Median	28	30	31
Q1	25	24	25
Q3	34	26	36

Supplementary Fig. S2 Obstructive breathing events and work of breathing. Results of studied 92 infants in supine-, side-, and prone-sleeping positions. The side sleeping position reduced the number of obstructive events, work of breathing, and 95th percentile levels of end-tidal carbon dioxide when compared with supine-sleeping.

$EtCO_2P_{95}$ 95th percentile level of end-tidal carbon dioxide, *OAHI* obstructive apnea-hypopnea index, *$ODI_{\geq 3}OAHI$* oxyhemoglobin desaturation index ≥3% from baseline after obstructive apnea or mixed apnea or obstructive hypopnea, *SpO_2 MinOAHI* minimum pulse oximeter hemoglobin saturation value following obstructive and mixed apneas, or obstructive hypopnea, *$TcCO_2P_{95}$* 95th percentile level of transcutaneous carbon dioxide.
